# High-value laboratory testing for hospitalized COVID-19 patients: a review

**DOI:** 10.2217/fvl-2020-0316

**Published:** 2021-09-21

**Authors:** Daniela Cihakova, Michael B Streiff, Steven P Menez, Teresa K Chen, Nisha A Gilotra, Erin D Michos, Kieren A Marr, Andrew H Karaba, Matthew L Robinson, Paul W Blair, Maria V Dioverti, Wendy S Post, Andrea L Cox, Annukka A R Antar

**Affiliations:** 1 ^1^Department of Pathology, Johns Hopkins University School of Medicine, 600 N. Wolfe Street, Baltimore, MD 21287, USA; 2 ^2^Department of Medicine, Johns Hopkins University School of Medicine, 733 N. Broadway, Baltimore, MD 21205, USA; 3 ^3^Austere environments Consortium for Enhanced Sepsis Outcomes, Henry M. Jackson Foundation, 6700 Rockledge Drive, Bethesda, MD 20817, USA

**Keywords:** CRP, COVID-19, d-dimer, high value care, IL-6, inpatient, laboratory test, labs, SARS-CoV-2, troponin

## Abstract

We present here an evidence-based review of the utility, timing, and indications for laboratory test use in the domains of inflammation, cardiology, hematology, nephrology and co-infection for clinicians managing the care of hospitalized COVID-19 patients. Levels of IL-6, CRP, absolute lymphocyte count, neutrophils and neutrophil-to-lymphocyte ratio obtained upon admission may help predict the severity of COVID-19. Elevated LDH, ferritin, AST, and d-dimer are associated with severe illness and mortality. Elevated cardiac troponin at hospital admission can alert clinicians to patients at risk for cardiac complications. Elevated proBNP may help distinguish a cardiac complication from noncardiac etiologies. Evaluation for co-infection is typically unnecessary in nonsevere cases but is essential in severe COVID-19, intensive care unit patients, and immunocompromised patients.

## Lay abstract

Doctors managing the complex care of individuals with COVID-19 need timely evidence to guide which lab tests to send to predict outcomes and prevent and treat COVID-19 complications involving the heart, blood clots, the kidney, and other infections that occur during the hospital course. Several lab tests such as IL-6, CRP and white blood cell subset counts may help predict the severity of COVID-19 during the patient’s hospital course if obtained when the patient first presents to the hospital. Other tests such as LDH, ferritin and AST are also associated with severe illness and mortality but have less evidence for their utility beyond IL-6, CRP and other tests. A test related to blood coagulation, d-dimer, is also associated with COVID-19 severity, and it may be used if the patient is suspected of having a blood clot. Two heart biomarkers – cardiac troponin and proBNP – may help doctors diagnose and manage heart-related complications of COVID-19. Patients in the hospital with COVID-19 may be susceptible to other infections, but testing for these is most useful in patients with severe disease, such as those in the intensive care unit. Specific recommendations for testing for viral, bacterial and fungal infections are presented here. The judicious use of laboratory testing can help identify patients at high risk for severe or critical COVID-19 and aid in prevention, diagnosis and treatment of common COVID-19 complications.

As thousands of people with COVID-19 are newly hospitalized each day [1], clinicians around the world are rapidly learning how to manage this complex disease. Several laboratory tests may help clinicians predict outcomes and prevent or diagnose complications in patients with COVID-19. Here we provide an evidence-based review of appropriate laboratory testing in hospitalized, symptomatic COVID-19 patients for the practicing clinician.

## Inflammatory markers

### IL-6

A wide range of cytokines and chemokines are involved in the pathophysiology of COVID-19, but several studies reviewed in this section demonstrate that IL-6 is perhaps one of the most useful biomarkers in predicting COVID-19 severity during hospitalization and can also distinguish severe COVID-19 from severe influenza infection [2–4]. IL-6 is a pro-inflammatory cytokine primarily released by monocytes, macrophages and dendritic cells in response to pathogens, cellular damage, or signaling from other pro-inflammatory cytokines [5]. IL-6 stimulation of hepatocytes leads to reduced production of albumin and cytochrome p450 and rapid release of hematopoiesis factors and acute phase proteins such as CRP [6]. Other effects of IL-6 include differentiation of B cells, naive CD4^+^ T cells, and increased vascular permeability [6]. The actions of IL-6 are mediated through binding to soluble or membrane-bound IL-6 receptors, the latter of which is the target of tocilizumab and sarilumab [7]. Tocilizumab and sarilumab are IL-6 receptor inhibitors approved to treat inflammatory diseases such as rheumatoid arthritis and to treat cytokine release syndrome following CAR T-cell therapy of hematologic cancers. Tocilizumab received emergency use authorization on 24 June 2021, for hospitalized COVID-19 patients ages 2 years and up who are receiving systemic corticosteroids and require supplemental oxygen, noninvasive or invasive mechanical ventilation, or extracorporeal membrane oxygenation [8].

IL-6 is significantly higher on admission in COVID-19 patients who eventually experience severe or critical illness than in those with mild or moderate disease [2,9–12]. The risk of clinical deterioration within 3 days of admission is correlated with increasingly higher admission IL-6 levels [13]. In addition, IL-6 levels are significantly higher in COVID-19 nonsurvivors than in survivors [2,14–16].

Limitations of broad IL-6 use include the current lack of assay standardization and variability by laboratory or institution. Therefore, it is challenging to use a strict cutoff to help prognosticate severity of disease in COVID-19. Two studies suggest that COVID-19 patients with a serum IL-6 >80 pg/ml are at high risk of respiratory failure and death [15–17]. The longer turnaround time of IL-6 testing also may limit its clinical utility. Given these limitations and lack of availability in certain settings, other inflammatory markers such as CRP and ferritin may be more useful in certain settings than IL-6.

### CRP

Serum levels of the acute-phase inflammatory protein, CRP, rise dramatically with infection, inflammation, or tissue injury. CRP is released primarily from hepatocytes, with IL-6 being the main inducer of *CRP* gene expression [18]. CRP activates the complement pathway and enhances phagocytosis of pathogens and apoptotic cells [18]. CRP production may be impaired in liver failure [19].

Most hospitalized patients with COVID-19 have elevated CRP levels [20,21]. However, extreme elevations of CRP are strongly associated with critical illness and mortality in COVID-19 [22,23]. In a prospective cohort study of 5279 patients in New York with COVID-19, a CRP >200 mg/l had a stronger association with critical illness than age or comorbidities (OR: 5.1; 95% CI 2.8 to 9.2) [24]. Like IL-6, a CRP level obtained on admission can be used as an independent predictor of COVID-19 disease severity during the patient’s hospital course [25]. In one study, COVID-19 patients with an admission CRP ≥65.1 mg/l were more likely to require high-flow oxygen, noninvasive or invasive mechanical ventilation, continuous renal replacement therapy or extracorporeal membrane oxygenation (OR: 8.9; 95% CI 1.0–74.2) [26]. A recent meta-analysis of 32 studies and >10,000 COVID-19 patients found that CRP was independently associated with higher risk of composite severe COVID-19 outcome (pooled-OR: 4.37; 95% CI 3.37–5.68) [27].

## Other laboratory tests associated with severe COVID-19

### Ferritin

Ferritin plays a central role in iron homeostasis and, like CRP, is an acute-phase reactant; its levels generally correlate with the degree of pathologic inflammation. Macrophages are likely a major source of ferritin release [28]. Extreme hyperferritinemia is a hallmark of secondary hemophagocytic lymphohistiocytosis or macrophage activation syndrome (MAS), a life-threatening hyperinflammatory disorder that can be triggered by infection. Severe COVID-19 may be complicated by MAS in some patients. In an intensive immunologic analysis of 28 COVID-19 patients with severe respiratory failure, seven (25%) had ferritin levels >4420 ng/ml and were classified as having MAS [29].

Ferritin is significantly elevated in severe cases of COVID-19 [14,30–34]. In one study of 229 COVID-19 patients, a ferritin level >750 ng/ml was an independent predictor of death (OR: 3.3; 95% CI 1.3–8.6) [35]. Another study of 252 COVID-19 patients found that those with a ferritin level ≥450 ng/ml were more likely to die (OR: 5.1; 95% CI 2.6–10.0). Two meta-analyses demonstrated a significant difference in ferritin levels between COVID-19 survivors and nonsurvivors as well as between severe COVID-19 and nonsevere COVID-19 [33,34].

### Lactate dehydrogenase

LDH is an enzyme present in nearly all cells; elevated levels in the bloodstream signify tissue damage. Elevated LDH is associated with an increased risk of severe COVID-19 and mortality in COVID-19 [27,36,37]. Conversely, LDH levels <425 U/l are associated with lower odds of intensive care unit (ICU) admission [37].

### White blood cell count & absolute lymphocyte count

Most patients with COVID-19 have a white blood cell count (WBC) in the normal range at presentation [20]. Severely ill COVID-19 patients may have a higher WBC than moderately sick patients. However, WBCs in severe COVID-19 patients are still significantly lower than in bacterial pneumonia [29]. Individuals with severe COVID-19 have a greater absolute neutrophil count, higher neutrophil frequency and higher neutrophil to lymphocyte ratio (NLR) [38–41]. Indeed, elevated neutrophil count and NLR, among other laboratory parameters, are predictors of severe disease [42,43]. Additionally, the trajectory of the neutrophil count and NLR early in the course of hospitalization also predict severity and mortality [44,45].

Approximately 60–90% of patients hospitalized with COVID-19 have lymphopenia at the time of presentation [20,21]. Lymphopenia, and specifically low CD4^+^ and CD8^+^ T cell counts, is associated with eventual development of critical illness and death [46,47]. The correlation of lymphopenia and severe COVID-19 and mortality has been supported by several meta-analyses [38,48]. Flow cytometry analyses of lymphocyte subsets in patients with COVID-19 demonstrated that besides T cells, B cells and NK cells were also diminished in severe COVID-19 [49]. Thus, WBC, neutrophil count and absolute lymphocyte count are associated with either COVID-19 diagnosis or severe outcomes.

### Transaminases & serum albumin

A vast majority of available reports on COVID-19 show only moderately increased ALT and AST levels [20,21] and moderately decreased serum albumin levels [47,50,51]. Additionally, a meta-analysis of 23 studies determined that mean liver function test values including ALT, AST and alkaline phosphatase, were normal, suggesting that most COVID-19 inpatients do not have liver function test elevation [52]. However, several reports suggest that elevated levels of ALT or AST are significantly higher in severe cases [47] and are associated with death [53,54], severe respiratory failure [29], and transfer to the ICU [53]. This association was evaluated by a meta-analysis with mostly hospitalized participants (n = 20,724) [55]. While heterogeneity between studies was high, there was an association between in-hospital mortality and elevated ALT (OR: 1.5; 95% CI: 1.1–2.0), AST (OR: 4.4; 95% CI: 2.7–7.2); and total bilirubin (OR: 7.8; 95% CI: 2.3–26.4) [55]. Of these, AST elevation precedes that of the others and has the strongest association with mortality [56]. While liver function tests abnormalities are generally only modest, they are associated with severe COVID-19 outcomes.

## Laboratory testing in treatment & prevention of venous thromboembolic disease

### D-dimer

SARS-CoV-2 infection results in a profound inflammatory prothrombotic state associated with a high risk of venous thromboembolism (VTE) [57]. Common laboratory abnormalities include elevated d-dimer, fibrinogen, factor VIII, von Willebrand factor and thrombocytosis [58]. Among these, d-dimer is the biomarker most closely associated with risk of VTE. Guidance statements from the International Society on Thrombosis and Haemostasis and from the American College of Chest Physicians, among other organizations, endorse VTE thromboprophylaxis in all hospitalized COVID-19 patients with acceptable bleeding risk [59,60].

Elevated d-dimer levels are associated with increased risk of severe COVID-19, VTE and mortality [61–63]. In a prospective ultrasound study of consecutive non-ICU patients, a d-dimer of <1570 ng/ml had a negative predictive value of 97.5% for VTE [64]. The American Society of Hematology endorses not ordering radiology studies for the diagnosis of pulmonary embolism if the d-dimer is normal in the context of low pretest probability in COVID-19 inpatients [8].

### Anti-Xa

COVID-19 is associated with heparin resistance [65,66]. This may be due to elevated levels of fibrinogen and other acute-phase reactants in COVID-19-associated inflammation, many of which bind heparin and antagonize its anticoagulant effects [67]. Given this, some clinicians monitor unfractionated heparin or low molecular weight heparin anti-Xa levels for those receiving therapeutic anticoagulation.

## Markers of cardiac injury & wall stress

COVID-19 carries significant cardiovascular morbidity and mortality and can manifest with an acute cardiovascular syndrome [68].

### Troponin

Cardiac troponin (cTn), a highly specific test for myocardial injury, can be measured by conventional or high sensitivity assays. In addition to its traditional role in indicating a Type 1 or Type 2 myocardial infarction, which both can occur in COVID-19, troponin elevation can reflect pre-existing cardiovascular disease (CVD) or accompany a number of other COVID-19 cardiovascular presentations including viral myocarditis, indirect cardiac injury from inflammation, stress cardiomyopathy, heart failure, pulmonary embolism or arrhythmias [69,70].

cTn is elevated in about 16% of hospitalized COVID-19 patients (95% CI: 9–27%) [71]. Elevated troponin in COVID-19 is detected more often in patients with a history of chronic CVD [72]. Importantly, studies and meta-analyses including large (>1000) numbers of COVID-19 inpatients demonstrate that elevated cTn is associated with increased risk of mortality, even after accounting for pre-existing CVD [73–78]. Patterns of increasing levels of cTn are also associated with increasing risk for mortality [77,79]. Elevated cTn is also associated with arrhythmia in COVID-19 inpatients [75,80].

Assessment of cTn on admission is suggested for hospitalized COVID-19 patients, particularly those with severe illness. For those with elevated troponin on admission, serial troponins and cardiac point-of-care ultrasound, if safe and available, are suggested to guide further imaging and management until cTn levels are stable or down-trending [72,81]. Troponin can be re-measured again among those with new ECG, hemodynamic or cardiac ultrasound changes and for those with signs or symptoms of clinical cardiac deterioration. In this setting, cTn measurement is a part of a comprehensive management strategy including triage, imaging, monitoring and involvement of consultants. COVID-19 inpatients with elevated troponin who have recovered should be referred for outpatient cardiology follow up for guidance on further diagnostic work up or longer-term management.

### ProBNP

ProBNP and its metabolite BNP are cardiac neurohormones that reflect ventricular wall stress from volume expansion and pressure overload [82] and have a well-established role in heart failure and various forms of myocarditis. BNP also helps distinguish cardiac and noncardiac causes of acute dyspnea [83–85].

ProBNP is elevated in approximately 10–15% of adult COVID-19 inpatients [73]. Even after adjusting for age, sex and comorbidities, an elevated proBNP value is associated with mortality (HR: 5.62; 95% CI: 3.99–7.93) [73,86,87], though not all studies report this [23]. One study noted that significantly rising proBNP levels were seen only in nonsurvivors [80].

ProBNP testing is useful for adult COVID-19 inpatients with signs or symptoms concerning for a cardiac complication that requires distinction from noncardiac etiologies. ProBNP or BNP testing is likewise useful in hospitalized children suspected of having multisystem inflammatory syndrome in children (MIS-C), and BNP has been identified as a key cardiac biomarker associated with severe MIS -C [88,89]. An elevated proBNP or BNP may prompt consideration of cardiac point-of-care ultrasound followed by transthoracic echocardiography when feasible given the association of proBNP with ventricular dysfunction and other urgent echocardiographic findings [90]. ProBNP measurement at hospital discharge in patients with elevated levels or a diagnosis of cardiomyopathy is reasonable, and may guide heart failure prognosis, treatment and follow-up strategies.

## Markers of kidney injury

### Basic metabolic panel & urine studies

COVID-19 inpatients are at increased risk of developing acute kidney injury (AKI) from a variety of mechanisms [91–93]. The incidence of AKI in patients hospitalized with COVID-19 is 20–50%, among which roughly 20% require kidney replacement therapy [94–96]. Potential etiologies for AKI include prerenal injury in the setting of volume depletion and high insensible losses, acute tubular injury from prolonged hemodynamic compromise and cytokine storm, thrombotic disease, glomerulonephritis, rhabdomyolysis and direct tubular injury from the virus [97,98]. AKI during hospitalization has been associated with significantly worse in-hospital mortality, with a meta-analysis of four COVID-19 cohorts demonstrating a HR of 3.08 (95% CI: 1.54–6.19) for mortality in patients with versus without AKI [99]. Additional evidence of kidney injury in the setting of COVID-19 include the presence of proteinuria and/or hematuria, which have been associated with worse short-term outcomes including in-hospital mortality [100]. Among survivors of COVID-19 hospitalization complicated by AKI, up to 35% did not recover to their baseline kidney function by the day of hospital discharge [92].

The initial evaluation of a hospitalized patient with COVID-19 includes a basic metabolic panel, urinalysis, urine output monitoring, assessment of volume status and evaluation of risk factors for kidney injury. Volume overload should be managed appropriately and exposure to nephrotoxins minimized [101]. Abnormalities on urinalysis require additional evaluation, including quantification of proteinuria or albuminuria and hematuria as indicated [93]. Worsening AKI, persistent volume overload, and/or electrolyte abnormalities refractory to medical management should prompt consideration for a nephrology consult.

## Testing for co-infection & secondary infection

### Viral & atypical bacterial respiratory testing

Viral and atypical bacterial (*Legionella* species, *Mycoplasma pneumoniae*, *Chlamydia pneumoniae* and *Chlamydia psittaci*) respiratory co-infection in COVID-19 inpatients is uncommon. Large case series reported a 0.2–5% incidence of respiratory viral or atypical bacterial respiratory co-infection in 2020 [20,102–104]. Unlike other co-infections, there is not a significant difference in rates of viral co-infection found in ICU versus all hospitalized COVID-19 inpatients [105]. Therefore, routine testing for these pathogens in COVID-19 inpatients should only be considered when there is high clinical suspicion and when test results will alter clinical management.

### Sputum cultures

Sputum cultures upon admission are useful primarily in severe COVID-19 pneumonia and in cases in which there is high clinical suspicion for typical bacterial respiratory co-infection (e.g., imaging findings are atypical for COVID-19). Respiratory sampling to test for ventilator-associated pneumonia in COVID-19 patients is discussed elsewhere [106]. The reported rates of co-infection, defined as a bacterial infection present upon admission for COVID-19, are between 1 and 8% in large case series and a meta-analysis [103,104,107]. Secondary infections arising 48 hours or more after admission for COVID-19 are more common, reported in 5–16% of COVID-19 inpatients [104,107–111]. The rates of secondary infection are higher in COVID-19 ICU patients compared with all hospitalized COVID-19 patients [105]. In two small prospective studies of 32 and 45 consecutive COVID-19 patients in ICUs with universal respiratory co-infection testing, between 40 and 44% of ICU patients had a bacterial respiratory co-occurring infection [112,113]. Secondary infection is associated with a higher risk of COVID-19-related mortality [108,114].

Sputum cultures are suggested for patients when they first meet the Infectious Diseases Society of America (IDSA)/American Thoracic Society (ATS) definition of severe community-acquired pneumonia. This includes people who require mechanical ventilation or vasopressors and selected others (those with three or more of the following: respiratory rate ≥30 breaths/minute, Pa_O2_/Fi_O2_ ratio ≤250, multilobar infiltrates, confusion/disorientation, uremia with blood urea nitrogen ≥20 mg/dl, leukopenia <4000 cells/μl, hypothermia with core temperature <36°C, or hypotension requiring aggressive fluid resuscitation) [115].

Sputum cultures are also suggested for inpatients empirically treated for methicillin-resistant *Staphylococcus aureus* (MRSA) or *Pseudomonas aeruginosa* infection to detect their presence [115]. Not all symptomatic COVID-19 inpatients will be able to produce expectorated sputum; we suggest against performing sputum induction for co-infection respiratory testing given the exposure risk and low clinical utility in this setting.

### Pneumococcal & *Legionella* urinary antigen

Pneumococcal urinary antigen testing (UAT) is used to identify *Streptococcus pneumoniae*, and therefore to justify narrowing of empiric antibiotic regimens in severe community-acquired pneumonia [115]. Pneumococcal UAT has a sensitivity of 74% in adults with pneumococcal pneumonia, which is higher than sputum and blood cultures [116]. *Legionella* UAT detects disease caused by *L. pneumophila* serotype 1. In the largest study of UAT in COVID-19, 0 of 249 pneumococcal UAT and 0 of 246 *Legionella* UAT returned positive, which was significantly fewer than in influenza patients [117].

Given relatively low rates of co-infection with these pathogens, we suggest not performing Pneumococcal or *Legionella* UAT except in severe pneumonia (see IDSA/ATS definition above). *Legionella* UAT may be useful in mild or moderate pneumonia when *L. pneumophila* is suspected based on epidemiologic factors [115].

### Blood cultures

Bacteremia is uncommon in non-ICU COVID-19 inpatients, and so we suggest that blood culture ordering practices not be altered specifically for COVID-19 inpatients. In a study of 88,201 blood cultures, the rate of true bacteremia among COVID-19 inpatients with blood cultures was 1.6%, significantly lower than that in COVID-19-negative inpatients with blood cultures (5.9%; p < 0.001) [118]. Bacteremia is found more commonly in critically ill COVID-19 patients than in mildly or moderately ill COVID-19 inpatients. A case series from two New York City hospitals reported bacteremia in 15/126 (11.9%) patients who required mechanical ventilation compared with 4/222 (1.8%) who did not [102].

Blood cultures are suggested for patients who meet the IDSA/ATS definition of severe community-acquired pneumonia; this includes people who require mechanical ventilation or vasopressors and selected others [115]. Blood cultures are also suggested for inpatients empirically treated for MRSA or *Pseudomonas* infection to detect their presence [115]. Blood cultures are also suggested when co-infection is suspected and the pretest probability of bacteremia or candidemia is moderate or high.

### Serum fungal markers & fungal respiratory sampling

Compelling evidence of pulmonary aspergillosis developing in people with severe COVID-19 infection, also known as COVID-19-associated pulmonary aspergillosis (CAPA) has emerged [119–123]. In a prospective ICU study, bronchoalveolar lavage galactomannan and Aspergillus PCR and serum galactomannan testing documented CAPA in 27.7% of patients; CAPA diagnosis was associated with high mortality [121]. A trend toward better outcomes in those with CAPA treated with antifungals suggests significance, similar to outcomes in people with severe influenza-associated aspergillosis [124].

We suggest infectious diseases consultation, consideration of chest imaging, and lower respiratory tract sampling (if safe and available) for culture, galactomannan testing, and *Aspergillus* PCR (if available) when CAPA is suspected, especially in COVID-19 inpatients with refractory respiratory failure and clinical signs such as refractory fever, new fever, hemoptysis, and worsening respiratory status, in concordance with the European Confederation for Medical Mycology consensus criteria statement [123]. Definitions of probable, possible, and proven CAPA based on host factors, clinical factors and mycologic evidence have been proposed [123]. Serum beta-D-glucan assay can be a helpful adjunctive diagnostic biomarker for aspergillosis in immunosuppressed hosts and may provide reasonable negative predictive value, but low specificity is a concern, especially in non-neutropenic patients and COVID-19 inpatients [125,126]. Similarly, serum galactomannan is positive in 20% or less of individuals with CAPA [127,128]. Given limited sensitivities of all serum-based biomarker assays noted in CAPA cohort studies, negative predictive value may be compromised, but positivity may be reasonably predictive of disease [129]. More data are needed on diagnostic assay performance in this setting, as much disease appears limited to the airways.

### Co-infection testing in COVID-19 patients with compromised immunity

Transplant recipients are at risk for concurrent diseases that may appear contextually similar to COVID-19 pneumonia and require additional therapies, such as cytomegalovirus pneumonitis and respiratory virus lower tract involvement. Other populations may experience concurrent non-infectious complications that require additional immunosuppressive therapies, such as lung transplant recipients who develop bronchiolitis obliterans syndromes and oncology patients recently treated with immune checkpoint inhibitors or chimeric antigen T cell therapies. In a large cohort of cancer patients with COVID-19, recent treatment with immune checkpoint inhibitors was associated with hospitalization and severe disease [130]. For these reasons, the clinician must maintain a high degree of diagnostic diligence for classic opportunistic infections in these populations and consider early involvement of infectious diseases consultants.

## Conclusion

A thoughtful and judicious approach to laboratory testing is critically important for front-line clinicians caring for inpatients with COVID-19 (Table 1). Tests such as IL-6 and/or CRP, d-dimer, cTn, complete blood count with differential, and complete metabolic panel are suggested upon admission as part of a comprehensive examination and workup for determining the appropriate level of care and prognostication. AST, neutrophil-to-lymphocyte ratio and IL-6 and/or CRP may have added value when trended at least once for prognostication. Creatinine and urine output should be monitored throughout the hospitalization. Urinalysis and further workup is indicated if renal injury is apparent. ProBNP should be sent if there is concern for a cardiac complication. Elevated cTn or proBNP may prompt further workup for cardiac complications of COVID-19 and consideration of cardiac point-of-care ultrasound, if available. d-dimer may have utility in ruling out pulmonary embolism in COVID-19 patients if the pretest probability is low. In immunocompetent patients, blood cultures, sputum cultures, and Pneumococcal and *Legionella* urinary antigen testing should be reserved for COVID-19 inpatients who meet IDSA/ATS definition of severe community-acquired pneumonia or for a few specific scenarios detailed above. Aspergillosis is a concern for COVID-19 patients in the ICU, and consultation with infectious diseases, imaging, respiratory sampling and serum beta-D-glucan and galactomannan are indicated when there is clinical suspicion for CAPA. Serum beta-D-glucan and galactomannan have poor negative predictive value, as many patients with CAPA will not have abnormal results.

**Table 1.** Laboratory testing utility and guidance in hospitalized COVID-19 patients.

Laboratory test	Utility in COVID-19 and associated diseases	Suggested context and timing of testing	Ref.
Biochemistry			
			
IL-6	Extreme elevation associated with clinical deterioration, severe or critical illness and mortality.	Admission (if available with reasonable turnaround time).	[2,3,9–16]
CRP	Extreme elevation associated with critical illness and mortality.	Admission, other times may or may not be useful.	[22–27]
Ferritin	Extreme elevation associated with severe illness and mortality.	Unclear added utility beyond other markers listed here.	[14,29–35]
LDH	Elevation associated with increased mortality.	Unclear added utility beyond other markers listed here.	[27,36,37]
AST and ALT	Extreme elevation associated with severe illness and mortality.	Admission, regular intervals during acute-care hospitalization.	[47,52–56]
Creatinine and BUN	Acute renal injury associated with mortality.	Admission, regular intervals during acute-care hospitalization.	[92–100]
Urinalysis	Proteinuria and hematuria associated with poor short-term outcomes including mortality.	Admission and upon new acute renal injury.	[93,100]
Cardiac troponin	Elevated and increasing levels associated with cardiac complications and mortality.	Admission, signs or symptoms of cardiac deterioration, new ECG or hemodynamic changes, new cardiac ultrasound changes.	[71–81]
ProBNP	Elevated and increasing levels associated with cardiac complications and mortality.	Signs/symptoms of cardiac complication of COVID-19 that require distinction from noncardiac etiologies. Upon discharge for those with cardiomyopathy and those with elevated levels upon admission.	[73,80,86–90]

**Hematology**			
Neutrophil count or neutrophil-to-lymphocyte ratio	Elevation associated with severe illness and mortality.	Admission, trend over time may be useful in predicting severity.	[38–45]
Absolute lymphocyte count	Lymphopenia associated with severe illness and mortality.	Admission, other times may or may not be useful.	[20,21,38,48,49]
WBC	Elevation associated with severe illness.	Admission, other times may or may not be useful.	[20,29]
D-dimer	Elevated levels associated with increased risk of severity, venous thromboembolism and mortality.	Admission and as indicated based on venous thromboembolic risk.	[8,58–64]
Anti-Xa	Some clinicians monitor anticoagulated patients with anti-Xa levels given COVID-19-associated heparin resistance.	Per local guidance.	[65–67]

**Microbiology**^†^			
Viral and atypical bacterial respiratory testing	Low (1–3%) reported incidence of co-infection with these pathogens.	Do not routinely test. Test only if there is high clinical suspicion for a specific pathogen and test results will alter clinical management.	[20,102–105]
Sputum cultures	Low incidence of typical respiratory bacterial co-infection in noncritically ill patients. Moderate incidence in critically ill patients.	Test when severe pneumonia is diagnosed (see IDSA/ATS definition in text). Test those empirically treated for MRSA or *Pseudomonas*. Testing in suspected ventilator-associated pneumonia discussed elsewhere.	[103–115]
Pneumococcal and *Legionella* urinary antigens	Extremely low incidence of positive urinary antigen testing in COVID-19.	Test when severe pneumonia is diagnosed (see IDSA/ATS definition in text). *Legionella* UAT may be useful when this pathogen is suspected based on epidemiologic factors.	[115–117]
Blood cultures	Extremely low incidence of bacteremia in non-ICU COVID-19 patients. Low-to-moderate incidence of bacteremia in critically-ill COVID-19 patients	Test when severe pneumonia is diagnosed (see IDSA/ATS definition in text). Test those empirically treated for MRSA or *Pseudomonas*. Test in cases of suspected co-infection in which pretest probability of bacteremia is moderate or high.	[102,115,118]
Fungal respiratory sampling	Moderate incidence of CAPA in intubated patients.	BAL galactomannan, culture and *Aspergillus* PCR^‡^ are suggested when CAPA is suspected.	[119–131]
Serum fungal markers	Beta-D-glucan may be useful for its negative predictive value in intubated patients. Galactomannan may be useful for its positive predictive value in intubated patients.	Do not routinely test in nonintubated patients. Test in patients at high risk for CAPA. CAPA requires respiratory sampling for diagnosis.	[123–129]

† Testing considerations for immunocompromised people hospitalized with COVID-19 are not included in this table. See text for discussion.

‡ *Aspergillus* PCR is not widely available in the United States.

ALT: Alanine aminotransferase; AST: Aspartate aminotransferase; BAL: Bronchioalveolar lavage; BUN: Blood urea nitrogen; CAPA: COVID-19-associated pulmonary aspergillosis; CRP: C-reactive protein; ICU: Intensive care unit; IDSA/ATS: Infectious Diseases Society of America/American Thoracic Society; LDH: Lactate dehydrogenase; MRSA: Methicillin-resistant s*taphylococcus aureus*; proBNP: Pro-B-type natriuretic peptide; UAT: Urinary antigen testing; WBC: White blood cell count.

Key populations such as children, especially those with MIS-C, are not covered in this text. Hospitalized children with COVID-19 are likely to have elevated CRP and LDH and leukocyte indices are less reliable [131,132]. Children with MIS-C have grossly elevated inflammatory markers, proBNP, troponin and d-dimer [133]. People living with HIV with suppressed viral loads who are hospitalized with COVID-19 have similar levels of CRP, IL-6, ferritin and neutrophil counts as HIV-negative individuals, though these laboratory values were lower in people living with HIV without suppressed viral loads [134,135]. Chronic hepatitis B in COVID-19 inpatients may predispose to abnormal liver transaminases, and chronic active hepatitis C may be associated with COVID-19 severity [136,137].

Here we have reviewed in detail whether and how specific laboratory test abnormalities are associated with poor prognosis and important complications from COVID-19 such as VTE, kidney injury, adverse cardiac events and co-infection.

## Future perspective

This review summarizes the literature regarding the utility and indications of various laboratory tests in hospitalized COVID-19 patients from the first year and a half of the COVID-19 pandemic. Already now there are multiple risk prediction tools that can reasonably predict critical outcomes based on vital signs, exam and lab tests obtained in the first 24 hours after presentation to the hospital or emergency room. We expect these risk prediction tools to improve and gain widespread use in the near future and thus narrow the potential laboratory tests used in risk prediction at admission and risk prediction during the course of hospitalization. Specific biomarkers heralding critical illness may yet be discovered, with the ultimate goal being a point-of-care home- or office-based test or panel of tests used for risk prediction, medication decision-making, and triage to the hospital in native infection and vaccine breakthrough infection.

**Executive summary**

- Inflammatory markers and biochemical tests associated with COVID-19 severity and/or mortality
  ○ IL-6
    ▪ IL-6 on admission and follow up is strongly correlated with severity and mortality in COVID-19
  ○ CRP
    ▪ CRP on admission is strongly associated with severity and mortality in COVID-19
  ○ Ferritin, lactate dehydrogenase and white blood cell count
    ▪ Also correlated with COVID-19 severity and mortality, though typically not as strong as IL-6 or CRP
  ○ Lymphocyte and neutrophil counts
    ▪ Low absolute lymphocyte count is correlated with COVID-19 severity and mortality
    ▪ Elevated neutrophil count or neutrophil-to-lymphocyte ratio also correlated with COVID-19 severity and mortality
  ○ AST/ALT
    ▪ AST > ALT associates with COVID-19 severity and mortality
- Testing in the treatment and prevention of venous thromboembolic disease
  ○ D-dimer is closely associated with risk of venous thromboembolism, COVID-19 severity and mortality
  ○ A normal d-dimer in context of low pretest probability is suggestive of no pulmonary embolism
  ○ Heparin resistance may be associated with COVID-19
- Markers of cardiac injury and wall stress
  ○ Cardiac troponin
    ▪ Indicates myocardial injury; is elevated in 10–25% of COVID-19 inpatients
    ▪ Elevated troponin is associated with mortality and cardiac complications of COVID-19
    ▪ Send troponin on admission; if elevated, obtain serial troponin levels and further evaluate
  ○ ProBNP
    ▪ Reflects ventricular wall stress; elevated in 10–15% of COVID-19 inpatients
    ▪ Send if concerned for a cardiac complication that requires distinction from noncardiac etiologies
    ▪ A key cardiac biomarkers associated with multisystem inflammatory syndrome in children
- Markers of kidney injury
  ○ Incidence of acute kidney injury is 20–50%
  ○ Creatinine, blood urea nitrogen, urine output monitoring should be assessed throughout the hospital course
  ○ Abnormalities on urinalysis require additional evaluation
- Testing for co-infection and secondary infection
  ○ Viral and atypical bacterial respiratory testing
    ▪ 0.2–5% incidence of co-infection with these pathogens
    ▪ Testing often does not change management
    ▪ Consider when clinical suspicion is high and when test results would alter management
  ○ Sputum cultures
    ▪ 1–8% incidence of any bacterial co-infection (present at admission)
    ▪ 5–16% incidence of any bacterial secondary infection (48+ h after admission); incidence is higher in intensive care unit (ICU) patients
    ▪ Send sputum cultures when patients meet Infectious Diseases Society of America (IDSA)/American Thoracic Society (ATS) definition of severe community-acquired pneumonia (CAP) or for specific situations discussed in text
  ○ Pneumococcal and *Legionella* urinary antigen
    ▪ Send urinary antigens when patients meet IDSA/ATS definition of severe CAP and when *Legionella pneumophila* is suspected based on epidemiologic factors
  ○ Blood cultures
    ▪ Bacteremia is uncommon in non-ICU patients (<2%)
    ▪ Send blood cultures when patients meet IDSA/ATS definition of severe CAP or for specific situations discussed in text
  ○ Serum fungal markers and fungal respiratory sampling
    ▪ COVID-19-associated pulmonary aspergillosis (CAPA) occurs in ICU patients
    ▪ Monitoring for CAPA is institution specific
    ▪ Clinical suspicion warrants infectious diseases consultation, imaging, and respiratory and serum testing
    ▪ Serum beta-D-glucan and galactomannan test sensitivity in CAPA is low, limiting negative predictive value
  ○ Immunocompromised patients
    ▪ Involve infectious diseases consultants and monitor for opportunistic infections
